# Exploring Upper Limb Malformation Associated With Cornelia De Lange Syndrome: A Clinical Case Report

**DOI:** 10.1155/crog/9963522

**Published:** 2026-07-31

**Authors:** Andrew Pratama Kurniawan, Gatot Abdurrazak, Aidrus Abdul Muthalib, Rachmat Dediat Kapnosa Hasani

**Affiliations:** ^1^ Department of Obstetrics and Gynecology, Faculty of Medicine, Universitas Indonesia, Jakarta, Indonesia, ui.ac.id; ^2^ Department of Obstetrics and Gynecology, Harapan Kita Hospital, Jakarta, Indonesia

**Keywords:** congenital, Cornelia de Lange syndrome, deformity, syndrome, upper extremity malformation

## Abstract

**Introduction:**

Upper extremity deformation may come in isolation, such as polydactyly, or be associated with a syndrome involving other body parts. Cornelia de Lange syndrome (CDLS) is a syndrome encompassing many abnormalities with some variation and a spectrum of severity. This case report presents a case of a baby with suspected CDLS.

**Case Presentation:**

A 28‐year‐old woman, Gravida 3 Para 2, came to the polyclinic referred because of her small gestational age and a possibility of congenital abnormality. The ultrasound examination showed upper extremity malformation with hypoplasia of the left radius and ulna, right ulna, agenesis of the right ulna, intrauterine growth restriction, and polyhydramnios. An elective Caesarean section was performed at 37 weeks of gestational age due to two previous Caesarean sections. Baby girl was born at 1446 g, with a body length of 39 cm and an Apgar score of 6/8. The baby had some features of CDLS, such as thick eyebrows, a short nose, a concave nasal ridge, a long indistinct philtrum, and a distinct upper extremity malformation.

**Discussion:**

CDLS was characterized by prenatal growth retardation, microcephaly, craniofacial abnormalities, hand or foot malformation, and hirsutism in the face. Prenatally, CDLS has some distinctive features in ultrasound, such as fetal growth retardation, craniofacial abnormalities, and limb malformation. CDLS has a broad spectrum of clinical presentation; its prognosis may vary based on the abnormality and the symptoms caused.

## 1. Introduction

Prenatal screening and diagnosis for congenital malformation are essential in providing consultation for the expectant couple. The finding of a congenital malformation during antenatal care may alter the treatment and care for the associated condition. Major congenital anomalies were affecting a total of 23.9 per 1000 births in Europe for 2003–2007 [1]. WHO estimated that 240,000 newborns died worldwide within 28 days every year due to birth defects [2]. Although upper extremity malformation is rarely lethal, it is one of the most common congenital disabilities. A study found that the overall prevalence of upper extremity abnormalities in the United States was 27.2 cases per 10,000 live births, with polydactyly as the most common defect (23.4 cases per 10,000 live births) [3].

However, upper extremity evaluation is commonly missed during regular examination. A usual biometry examination does not require the examiner to measure the humerus, radius, or ulnar length. Thus, some upper extremity abnormalities may be missed and discovered much later [4]. It is crucial to determine whether the abnormalities found are isolated in the upper extremity or associated with a syndrome. Examples of syndrome that associated with upper extremity abnormalities are amniotic band syndrome, Poland syndrome, Klippel–Trenaunay–Weber syndrome, Rubenstein–Taybi syndrome, and Cornelia de Lange syndrome (CDLS) [3, 5]. We would like to present the case of upper extremity abnormalities that were referred because of fetal growth retardation. The baby was later found to be associated with CDLS.

## 2. Case

A 28‐year‐old woman, Gravida three Para two, came to the polyclinic because of her small weight for gestational age and a possibility of congenital abnormality. She was 34 weeks pregnant based on the first‐trimester ultrasound and her last menstrual period (LMP). The fetus was noticed to have restricted growth since 28 weeks of gestational age. There were no remarkable notes in the patient′s past illness or medication. Her first pregnancy ended in a Caesarean section (C‐section) due to placenta previa and delivered a full‐term baby boy weighing 2800 g. In the second pregnancy, she underwent another C‐section because of premature rupture of the membrane and delivered a boy weighing 2700 g.

**Figure 1:**
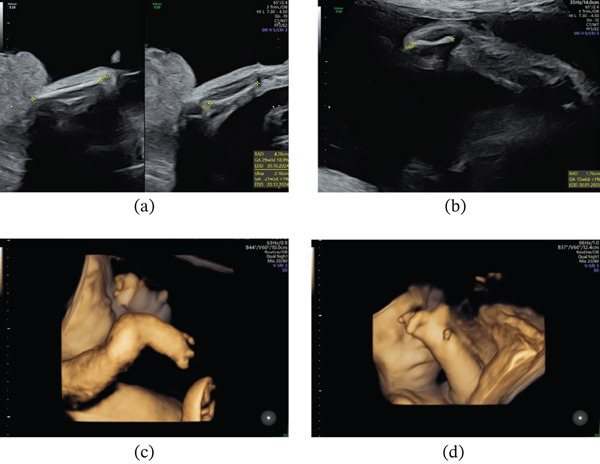
Upper extremity deformities. (a) Hypoplasia of the left radius and ulna, (b) agenesis of the right ulna and hypoplasia of the right radius, (c) malformation of the upper extremity with an abnormal range of motion of the elbow, and (d) total of only three severely deformed fingers on the right hand.

From physical examination, the patient was hemodynamically stable with normal general examination. Her fundal height was 30 cm, with a normal fetal heart rate range. However, the fetal part was hard to palpate. The ultrasound examination showed a singleton live intrauterine fetus with multiple congenital abnormalities. We found upper extremity malformation with hypoplasia of the left radius and ulnar bone, hypoplasia of left radius bone, aplasia of right ulnar bone, and abnormal finger counts (Figure 1). There was no abnormality in the central nervous, gastrointestinal, urinary, and cardiovascular systems. The fetus′s biometry was also extremely small, as her biparietal diameter, head circumference, abdominal circumference, femur, and humerus length were below one percentile. The fetus was also polyhydramnios with a single deepest pocket of 9.4 cm. The umbilical cord was unremarkable. Doppler ultrasonography results for the systolic/diastolic ratio for the umbilical artery are increased based on the gestational age. Furthermore, the ductus venosus and medial cerebral artery flow were within normal limits. Since the gestational age is near term, we planned the follow‐up examination for another 2 weeks. In the follow‐up examination, there was an increase in fetal weight. Fetal biometry and Doppler results were similar to her last result, with no additional congenital abnormalities found.

**Figure 2:**
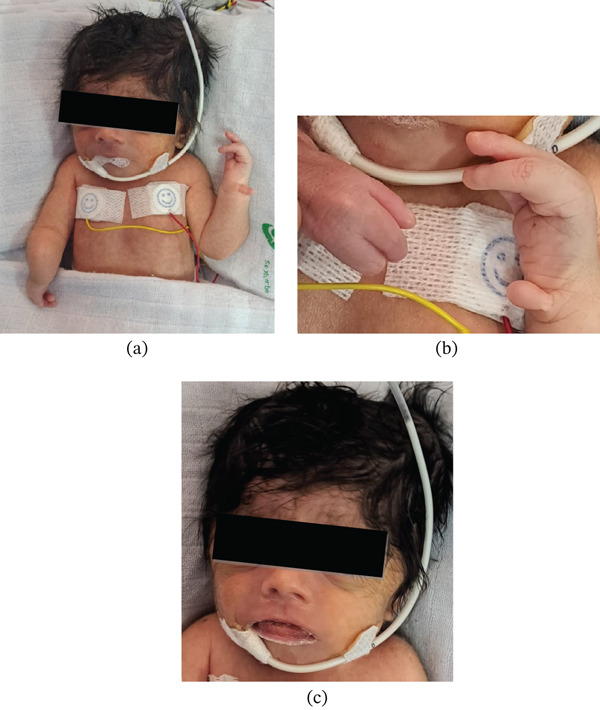
Macroscopic anomalies of the baby. (a) Anterior view of the upper body. The hands are small with oligodactyly. (b) The right hand had three fingers, whereas the left hand had four well‐developed fingers and a short fifth finger. (c) Facial abnormalities with thick eyebrows and synophrys, concave nasal ridge, long indistinct philtrum, and thin upper lip with downturned corners of the mouth.

**Figure 3:**
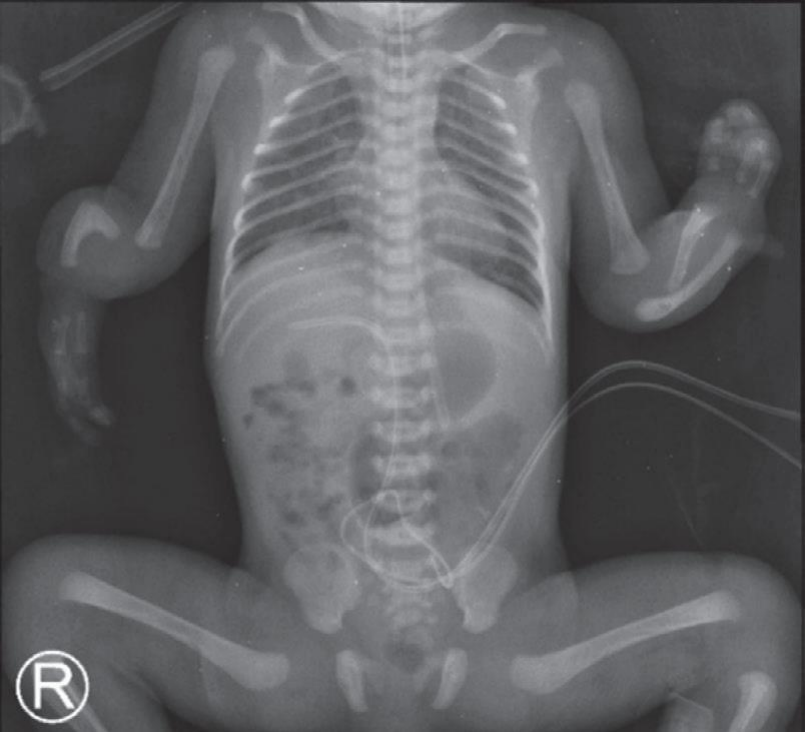
Babygram shows deformity in both upper limbs in the antebrachial region with oligodactyly.

An elective C‐section was performed at 37 weeks of gestational age due to two previous C‐sections. She delivered a baby girl of 1446 g, with a body length of 39 cm and an Apgar score of 6/8. The baby was unstable with chest retraction and was quickly given continuous positive airway pressure by the perinatology team. A further examination revealed that the baby had some features of CDLS. Some obvious signs included thick eyebrows, a short nose, a concave nasal ridge, a long indistinct philtrum, malformation of the lower arm, and a few dysmorphic fingers (Figure 2). The chest X‐ray and head ultrasonography were within normal limits. The baby was diagnosed with classic CDLS since it fulfilled the scoring criteria. X‐ray examination showed a deformity on the bilateral antebrachial with deformity on the right radius bone, aplasia of the right ulnar bone, and hypoplasia of the left radius and ulnar bone, with bilateral syndactyly (Figure 3). Molecular testing is needed for a definitive diagnosis. However, due to limited resources of diagnostic capabilities, a sample needs to be sent to a better genetic laboratory testing elsewhere, which the parents disagree with.

The parents had been given oral consent for academic publication purposes.

## 3. Discussion

This case report would like to highlight the importance of prenatal detection of upper extremity anomalies. However, despite the advances in ultrasound, the sensitivity remains poor. In a study, the sensitivity to detect upper extremity anomalies was 42%, with the highest sensitivity when it affected the whole upper extremity rather than digital anomalies [4]. Overall sensitivity is also higher when associated with a syndrome or another congenital abnormality rather than isolated malformations. Some factors may contribute to this low sensitivity in detecting anomalies in the upper extremities [4]. First, the upper extremity evaluation is often a cursory examination. Bone measurement of the upper extremities is not essential for fetal biometry calculations. Thus, routine screening for upper extremity abnormalities tends to be forgotten and has a high false‐negative rate.

Up until now, there has been no standardized protocol for limb‐focused screening. It is recommended that anomalous limb malformation be screened to detect abnormal size, shape, number of digits, and alignment. Since ultrasound evaluation is a real‐time examination, thus it is capable of detecting possible movement abnormalities. The ideal time to visualize the upper extremity is in the late first and early second trimesters since the fingers tend to be extended and abducted [4, 6]. This diagnostic challenge of upper extremity malformation could be addressed by the use of 3D ultrasound. The use of 3D ultrasound in this case helps in visualizing the upper extremity malformation clearly. Therefore, we recommend that the finding of reduced intrauterine fetal growth and low percentile of femur length should prompt an examination of other long bones and extremity anomalies.

The main prenatal features of CDLS are a growth‐restricted fetus, limb abnormalities, and some craniofacial abnormalities [7]. Limb abnormalities that may arise are often asymmetrical in the distal part of the upper limbs, such as micromelia, syndactyly, clinodactyly of the fifth finger, and oligodactyly [7]. In some severe cases, hypoplasia or agenesis of the radius or ulna could develop, as in this case. Notably, the short and almost rounded first metacarpal bone is one of the diagnostic indicators for CDLS. The craniofacial features, perhaps, are better evaluated with 3D ultrasound reconstruction to reveal a smooth, convex philtrum, an upturned nose with anteverted nostrils, a thin upper lip, micrognathia, and low‐set ears. Hypertrichosis is one of the main hallmarks of CDLS, manifesting in long eyelashes, arched eyebrows, and synophrys. Other malformations that are not exclusive to CDLS are congenital heart defects (coarctation of the aorta, pulmonary stenosis, tetralogy of Fallot, and atrial or ventricular septal defects) and genitourinary anomalies (hypoplasia, cryptorchidism, renal cyst, or hypoplasia) [8]. Prenatally, we have yet to identify the facial anomaly, but there are no other associated anomalies in this case.

The chromosomal disorder must be suspected if two or more anomalies are found. Prenatal invasive testing, such as chorionic villus sampling (CVS) or amniocentesis, could be done to investigate. CVS could be done earlier, within 10–14 weeks of gestational age, and amniocentesis for an older gestational age when the amniotic sac is more prominent, usually more than 15 weeks of gestational age [9]. Nevertheless, CDLS usually reveals a normal karyotype [8]. But, antenatal invasive testing could exclude some karyotyping anomalies, although noninvasive prenatal testing could also be done using the maternal serum to some extent. We chose not to perform any invasive prenatal testing since the patient was near term, around 34 weeks of gestational age, and there was no added value or fetal interventions that could be done to repair the condition [7]. Postnatal molecular and genetic testing is a better option in this case.

CDLS nowadays has a wide range of disease presentations and spectrums. A consensus was developed on classifying all clinical varieties and genetic heterogeneity in the spectrum of the disease [10]. The consensus divided the characteristics into two main features (Table 1). The cardinal features have 2 points added per feature, and the suggestive features have 1 point added per feature [10]. Classic CDLS was defined by ≥ 11 points, with three cardinal features. Nonclassic phenotype scored between 9 and 10 and has two cardinal features. When the score falls between 4 and 8 with one cardinal feature, molecular testing for CDLS is indicated. On the other hand, a score of < 4 points is insufficient to indicate molecular testing for CDLS. In our case, the patient has all of the cardinal features except congenital diaphragmatic hernia, with suggestive features of prenatal growth retardation, microcephaly, small hands, and a short fifth finger with a total score of 14, equal to classic CDLS.

**Table 1:** Diagnostic algorithm as suggested by the consensus to diagnose and classify CDLS [10].

Cardinal features	Score	Patient′s score
Meeting of the medial eyebrows in the midline and/or thick eyebrows	2	2
Short nose, concave nasal ridge, and/or nose with an upturned tip	2	2
Long and/or smooth philtrum	2	2
Thin upper lip and/or downturned corners of the mouth	2	2
Presence of fewer than the normal number of fingers and/or absence of fingers or toes from birth	2	2
Congenital diaphragmatic hernia	2	—
Suggestive features		
Global developmental delay and/or intellectual disability/learning disability	1	—
Prenatal growth retardation (restricted growth prior to birth)	1	1
Postnatal growth retardation	1	—
Microcephaly	1	1
Small hands and/or feet	1	1
Short fifth finger	1	1
Abnormally increased hair growth	1	—

Although a malformation in the upper extremities is noticed prenatally, there is a need to screen for other congenital malformations, such as heart malformation (no specific malformation associated), palate, eye, central nervous system (seizure), genitalia (cryptorchidism, micropenis, and hypospadias), congenital diaphragmatic hernia, and in the gastrointestinal system (intestinal malrotation) [7]. Although screening will provide no added value in CDLS diagnosis, it determines its severity and the need for other specific treatment. In this case, we performed echocardiography, a head ultrasound, and a babygram. We also found an open foramen ovale during echocardiography and will follow up on its severity and closure.

The prognosis of CDLS patients varies based on the disease phenotype [7]. A milder type of CDLS with mild to borderline psychomotor delay, less severe postnatal growth deficiency, and little or no major malformation may be associated with a good prognosis. Conversely, the classical type of CDLS has a worse prognosis than its counterpart. A prognostic study found that the risk of severe intellectual disability is higher in a patient with growth retardation, heart or limb malformation, moderate–severe sensorineural hypoacusis, NIPBL, or SM1CA mutation [11]. Since our patient has limb malformation with classical CDLS features, the prognosis of the patient is high to experience intellectual disability. Growth improvement in the children should be monitored with the CDLS growth curve, and supportive treatment should be given for the patient′s disability.

## 4. Conclusion

Upper extremity malformation is less noticeable and commonly missed. A thorough examination with ultrasound is needed to screen for anomalies from the proximal to the distal upper limb. 3D ultrasound helps diagnose upper extremity anomalies. The hallmark features of CDLS are Intrauterine growth restriction, craniofacial malformation, and upper extremity malformation. It is a rare syndrome that can affect other system organs and is caused by a gene mutation responsible for the cohesin pathway and complex. The prognosis of CDLS worsens in cases with severe psychomotor delay, growth retardation, and severe malformation.

## Funding

No funding was received for this manuscript.

## Disclosure

All authors have read and approved the final version of the manuscript and had full access to all of the data in this study and take complete responsibility for the integrity of the data and the accuracy of the data analysis. The corresponding author affirms that this manuscript is an honest, accurate, and transparent account of the study being reported; that no important aspects of the study have been omitted; and that any discrepancies from the study as planned (and, if relevant, registered) have been explained.

## Conflicts of Interest

The authors declare no conflicts of interest.

## Data Availability

The authors confirm that the data supporting the findings of this study are available within the article.
